# Photometric evolution unveils debris generation process of rocket bodies in geostationary transfer orbits

**DOI:** 10.1038/s41598-025-27947-1

**Published:** 2025-12-19

**Authors:** Qingwei Qiao, Yiding Ping

**Affiliations:** 1 Purple Mountain Observatory, Chinese Academy of Sciences, Nanjing, China; 2 School of Astronomy and Space Science, University of Science and Technology of China, Hefei, China

**Keywords:** Aerospace engineering, Environmental impact, Space physics

## Abstract

The study of the long-term evolution of space debris is critically important for understanding debris generation mechanisms and, consequently, for the protection of orbital resources. Here we present a photometric survey of the CZ-3 series rocket bodies in Geostationary Transfer Orbit (GTO), revealing that their standardized brightness exhibits a multi-stage variation pattern over an in-orbit timespan of 41 years. Specifically, the brightness initially decreases, then rapidly increases, followed by a subsequent decrease that eventually stabilizes. Based on our understanding of the CZ-3 rocket bodies’ multilayer surface structure, we propose that the shedding of surface materials caused by space weathering is the primary driver behind these brightness variations. A similar evolutionary trend is observed in the Atlas V series rocket bodies. Based on this mechanism, we also explore the potential origins of previously unidentified debris in GTO reported in earlier studies.

## Introduction

The amount of space debris has rapidly increased with the development of human space activities, leading to a corresponding rise in the collision risks for spacecraft in orbit. Due to the extremely high velocities involved, such collisions could have catastrophic consequences. The further spread of debris may trigger the ”Kessler Syndrome”^1^, a cascading collision effect that severely threaten orbital resources. Therefore, studying the evolution of space debris is of great importance for understanding the mechanisms of debris generation, and consequently for developing mitigation and protection strategies. Rocket bodies, which are the remnants of rocket upper stages left after launch events, represent a significant category of space debris. They are widely distributed across various orbits, from Low Earth Orbit (LEO) to Geostationary Orbit (GEO), and as of 2024, they account for approximately 6.0% of trackable space objects, yet their total mass represents a substantial 30.6%^2^. Due to their large mass and complex structures, and their tendency to tumble uncontrollably once in orbit, rocket bodies are particularly vulnerable to effects such as gravity gradients, solar radiation pressure, and atmospheric drag. These effects can lead to instability in attitude, structure, and orbit, increasing the likelihood of collisions and the generation of additional debris. As of May 2022, a total of 87 rocket body fragmentation incidents had been recorded^3^, primarily caused by collisions with other objects or explosions due to residual propellant, and an additional six rocket body fragmentation events have been reported by the NASA Orbital Debris Program Office since then^4^. These fragmentations have generated substantial amounts of space debris, and statistics show that, as of 2024, debris from rocket body fragment events accounts for 6,271 trackable fragments, or 28.2% of the total^2^. Therefore, the study of rocket bodies is a crucial part of space debris research.

Rocket bodies exposed to the space environment are influenced by various effects, including extreme temperature cycling, micrometeoroid impacts, vacuum outgassing, high-energy particle radiation, and atomic oxygen erosion^5^. Researchers can analyze the effects on rocket bodies using ground-based or in-orbit experiments^6–8^. However, while ground-based simulation experiments are often unable to fully replicate the actual complex space environment, data from short-term in-orbit experiments are typically insufficient to reveal the long-term evolutionary patterns of spacecraft materials. As a result, a comprehensive understanding of the evolution mechanisms of rocket bodies over long time scales remains lacking. Ground-based optical observations can indirectly indicate changes in the materials and structures of space objects^9^, overcoming the above limitations of ground simulations and in-orbit experiments. However, a challenge in ground-based optical observations is that it is hard to continuously observe the same rocket body with the same equipment over long periods in practice. Additionally, data from different observation epochs are often difficult to compare directly due to variations in observing conditions. To address this problem, we introduce a methodology commonly used in galactic cosmology^10^, which involves observing targets of the same type at different ages to uncover their evolutionary patterns.

Here, we used an optical telescope to observe 23 CZ-3 series rocket bodies in several months. These rocket bodies feature a consistent design, with the oldest one having been in orbit for over 41 years. Observing them provides valuable insight into the changes of these rocket bodies over time, making them ideal samples for studying the long-term evolutionary patterns. We found the relationship between the standardized brightness of these rocket bodies and their time in orbit, revealing a distinct five-stage variation pattern. A similar pattern of brightness variation was also observed in another series of rocket bodies. We believe that the multi-stage changes in brightness result from the degradation and shedding of surface materials caused by space weathering. This theory helps explain the High Area-to-Mass Ratio (HAMR) space debris previously detected in GTO^11,12^, inspiring new approaches for space debris identification and source tracing.

## Samples

**Fig. 1:**
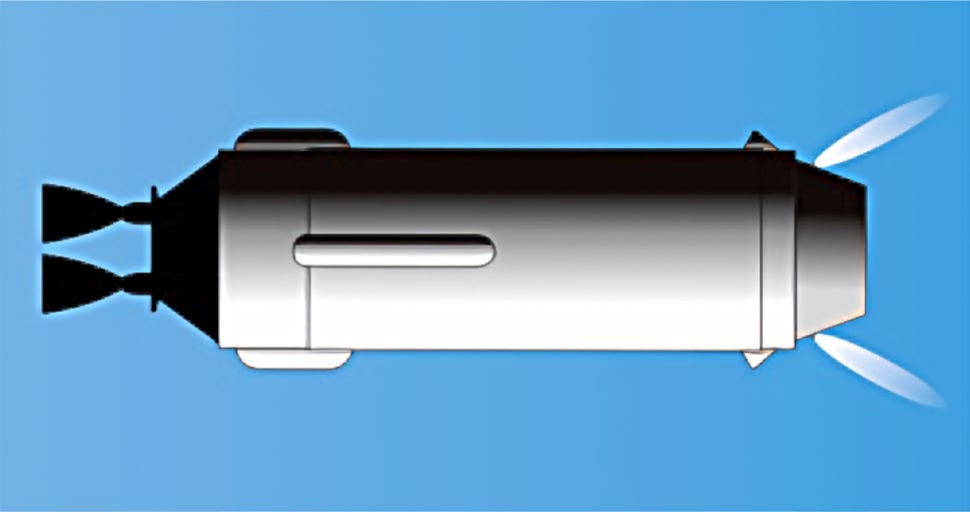
Artist rendition of a CZ-3 series rocket body: The cylindrical body is 12.375m long and 3m in diameter^13^, coated with a highly reflective white thermal control layer.

We selected two series of rocket bodies for observation. Group 1 comprises the CZ-3 series rocket bodies, including CZ-3 R/B, CZ-3A R/B, CZ-3B R/B, CZ-3C R/B, which are remnants of the core third stages of the Long March 3 series rockets, one of China’s main launch vehicles. These rocket bodies typically operate in GTO, and share almost the same design(Fig. 1). This series was chosen for study due to its advantages: (1) consistent configuration: All derivative models inherit the geometric dimensions and surface treatment processes of the base model, meaning differences in observational properties between individual rocket bodies are attributed to different evolutionary points in the evolutionary timeline; (2) long and continuous time span: Since 1984, more than 50 such objects have been recorded, with a time span of 41 years and a relatively uniform distribution, facilitating the identification of patterns in the observational data; (3) stable orbital characteristics: Most targets are located in GTO, minimizing variations in orbital environments and ensuring relatively uniform evolutionary paths.

Group 1 consists of 23 CZ-3 series rocket bodies, as shown in Table 1. These 23 targets were selected because they were observable by MEET and at least one complete light curve could be obtained during the observation season. The reason for requiring at least one full light curve will be explained in “Methods”. The orbital inclinations of these targets mostly range from 20° to 30°, with 5 exceeding 50°. Most of the targets have a perigee of several hundred kilometers, and their apogee lies between 10,000 and 40,000 km. The oldest rocket body, NORAD ID 14900, was launched in 1984, while the most recent one was launched in late 2024. Therefore, Group 1 covers a 41-year span with a relatively uniform time distribution. This comprehensive coverage allows for an accurate representation of the rocket body in-orbit history and provides insights into the evolutionary patterns of this rocket family.

**Table 1:** Main parameters of the selected CZ-3 series R/Bs.

ID	Name	Launch	Observe	Perigee^1^	Apogee $^\textrm{a}$	Number$^\textrm{b}$	Incl	Distance
				(km)	(km)		(degrees)	(km)
14900	CZ-3 R/B	1984-04-08	2024-11-18	456.97	34661.77	3	30.36	17586
16528	CZ-3 R/B	1986-02-01	2024-12-11	504.30	34936.55	6	30.79	19511
20474	CZ-3 R/B	1990-02-04	2025-03-18	294.03	25953.36	2	30.47	22259
26383	CZ-3A R/B	2000-06-25	2025-03-13	240.38	19317.94	2	27.21	8656
29517	CZ-3 R/B	2006-10-28	2024-12-25	520.49	10054.06	4	28.24	7000
33464	CZ-3A R/B	2008-12-23	2024-12-12	401.59	34817.30	6	24.85	14070
34780	CZ-3C R/B	2009-04-14	2024-12-18	325.31	32230.81	6	20.36	30479
39158	CZ-3B R/B	2013-05-01	2024-12-18	201.81	23530.18	2	26.45	18661
40368	CZ-3A R/B	2014-12-31	2025-03-13	299.79	36253.91	1	24.30	29464
41435	CZ-3A R/B	2016-03-29	2024-12-14	1700.15	32752.18	3	55.11	34042
41587	CZ-3B R/B	2016-06-12	2024-12-09	175.85	21252.77	3	19.36	16324
42663	CZ-3B R/B	2017-04-12	2025-03-18	232.34	30335.97	4	20.67	31375
43110	CZ-3B R/B	2018-01-11	2024-12-11	447.47	17516.19$^\textrm{c}$	7	54.95	18172
43649	CZ-3B R/B	2018-10-15	2024-12-27	491.22	17131.35$^\textrm{c}$	2	54.94	17364
44077	CZ-3B R/B	2019-03-31	2025-02-22	176.78	18117.22	4	26.90	19078
44205	CZ-3B R/B	2019-04-20	2024-12-09	177.65	17021.07	2	28.31	17394
45808	CZ-3B R/B	2020-06-23	2025-03-18	236.83	27365.84	1	28.22	28790
49331	CZ-3B R/B	2021-10-24	2024-11-18	313.27	33965.91	5	28.58	21593
52256	CZ-3B R/B	2022-04-15	2024-11-18	187.69	22835.85	2	28.17	19512
55687	CZ-3B R/B	2023-02-23	2024-12-27	208.47	29088.03	4	27.71	21456
59707	CZ-3B R/B	2024-05-09	2024-12-27	145.72	17647.25$^\textrm{c}$	4	53.23	18520
59916	CZ-3B R/B	2024-05-30	2024-12-11	189.68	32421.37	2	26.99	33705
62189	CZ-3B R/B	2024-12-03	2024-12-27	146.35	39762.63	2	51.35	25702

$^\textrm{a}$ The perigee altitude is calculated from the TLE data used for the observations and results may vary from date to date. The distance units used in this table are all km.

$^\textrm{b}$ This ”Number” means the number of period in our observed light curve.

$^\textrm{c}$ The target orbit for 43110 and 43649 is MEO. The target orbit for 59707 is MTO.

We found that the standardized brightness of the rocket bodies in Group 1 follows a distinct pattern over their in-orbit time. To validate this pattern, we extended our observations to another family of rocket bodies, the Atlas V Centaur R/B, which are primarily located in GTO (Table 2), as Group 2. This model of rocket upper stage also has an aluminum alloy main body and a multi-layer insulation structure, similar to the design of the CZ-3 series rocket bodies.

**Table 2:** Main parameters of the Atlas V Centaur R/Bs.

ID	Launch	Observe	Perigee	Apogee	Number	Incl	Distance
			(km)	(km)		(degrees)	(km)
27853	2003-07-17	2025-04-03	3763	34791	2	17.53	36165
34714	2003-07-17	2025-04-03	499	62339	8	20.87	56045
36396	2010-02-11	2025-04-03	2405	33488	2	29.38	31510
39071	2013-01-31	2025-04-03	4110	34685	1	26.18	36563
40295	2014-10-29	2025-04-03	20646	21320	3	56.89	21789
41623	2016-06-24	2025-04-03	3653	35184	4	18.68	34956
44483	2019-08-08	2025-04-11	13949	35217	4	20.36	26160
45466	2020-03-26	2025-04-03	10712	35447	2	7.07	21847
46919	2020-11-13	2025-04-03	10551	11003	1	58.92	10857
49819	2021-12-07	2025-04-11	36116	38660	2	3.34	40046

## Instruments

**Fig. 2:**
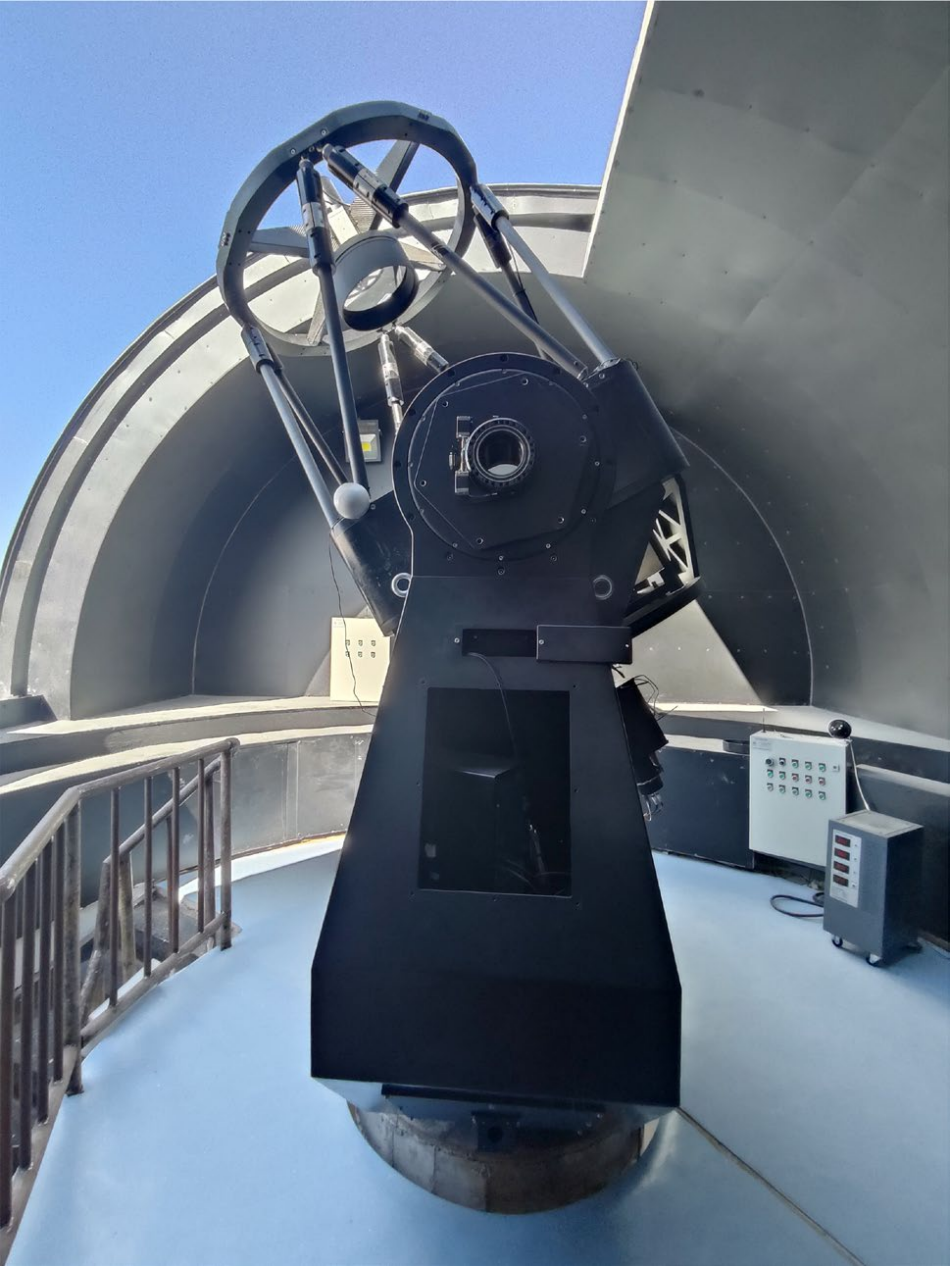
MEET in its dome. This telescope has an 80 cm aperture and adopts a Ritchey-Chrétien optical structure (focal ratio f/7). It has one Cassegrain focus and two Nasmyth foci. A QHY600 scientific-grade back-illuminated CMOS camera is configured at one of the Nasmyth foci. It has a field of view of $22.1'\times 14.8'$, and a 5-position filter wheel is installed at this focus.

We performed photometric observations with the 80-cm aperture Multi-Equipment Experimental Telescope (MEET) of the Purple Mountain Observatory(PMO) (Fig. 2). This telescope is located on Saishiteng Mountain, Lenghu, Qinghai Province (38°35’ N, 93°53’ E, elevation 3850 m)^14^. It was built as a pioneering instrument for the development of larger telescopes of PMO. The telescope is equipped with a high-precision dual-axis direct-drive system, providing good pointing and tracking accuracy. With the help of a QHY600^15^ CMOS camera at one of the Nasmyth foci, it can achieve fast tracking and photometry of rapidly moving GTO targets.

## Methods

We employed differential photometry to minimize the effects of atmospheric extinction on magnitude measurements. Due to variations in rocket body attitude and position during observations, the apparent magnitudes cannot be directly compared and must be normalized to a standardized brightness. The standardization process involves two main corrections applied to the raw photometric data. The first is the slant range correction, which adjusts all measured brightness values to a fixed slant range of 10,000 km, thereby eliminating the brightness attenuation caused by variations in the observer-target distance. Second, an attitude correction standardizes the brightness to a fixed viewing geometry, specifically with both the solar incidence angle and emergence angle set to 45°. This correction does not model the physical reflectance of the surface but provides a common geometric reference to ensure comparability among different observations. The attitude correction requires knowledge of the target’s maximum brightness within its light curve cycle. Therefore, it is essential to capture at least one complete light curve during observations to enable accurate correction.

### Observation

**Fig. 3:**
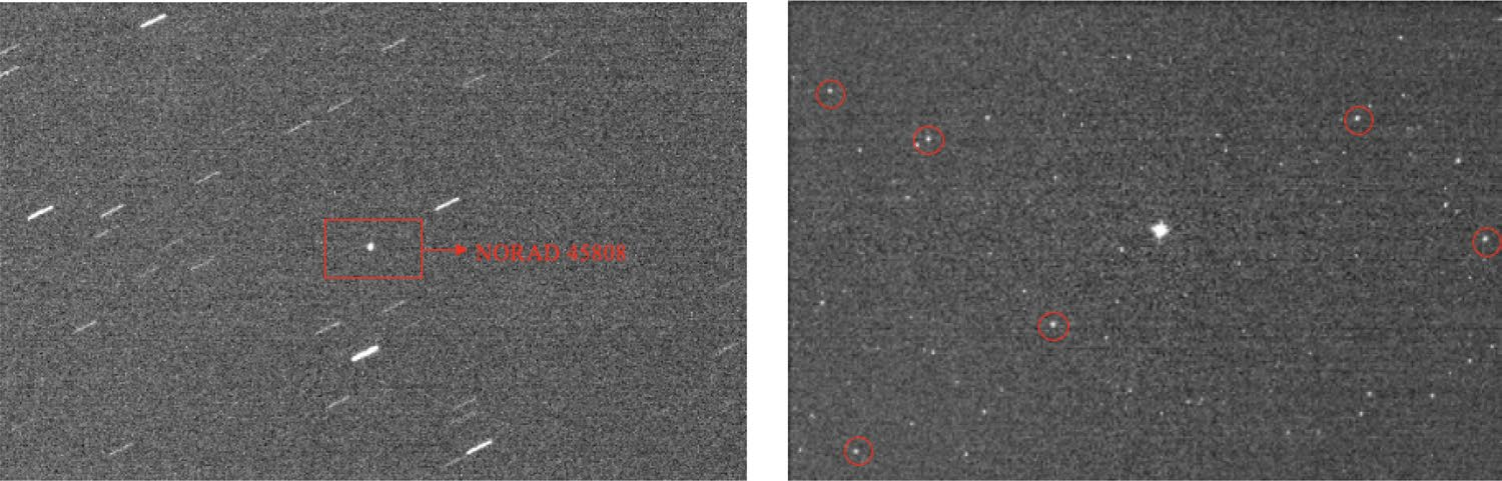
Typical observational images of the rocket body. Left: Target image acquired in tracking mode, where the rocket body appears as a point source while background stars are elongated. Right: Image of reference stars captured in sidereal tracking mode at nearby sky field after tracking the object, with selected reference stars indicated by red circles.

Observations were conducted in target tracking mode. This mode elongates the images of background stars (Fig. 3), thereby reducing the photometric accuracy of reference stars. So we ceased tracking immediately after acquiring the target’s light curve and subsequently captured standard photometric stars within the same or nearby fields using identical exposure settings. To minimize photometric errors caused by significant positional shifts, we preferentially observed near apogee, where their apparent motion is minimal. This strategy is particularly important because capturing a complete light curve in GTO often requires extended observation times, sometimes up to several tens of minutes. No filters were used during observations to maximize photon throughput and enhance the Signal-to-Noise Ratio (SNR), as the primary focus of this study was on overall brightness variations rather than spectral characteristics.

### Image reduction

We first performed basic astronomical image reduction for all captured images. During observations, camera cooling was enabled, with the cooling temperature set to -20 °C. The dark current of the QHY600 at -20°C is low, at 0.0022 e-/pixel/sec^15^, and the exposure time is 4 s, so the effect of dark current on the images was not considered. The QHY600 camera is configured with an overscan region. Bias correction was applied to all images using the column-averaged value of the overscan area as the background value. By taking twilight flats, we performed flat-field correction on the images of all targets and standard stars to improve photometric accuracy.

### Photometry

**Fig. 4:**
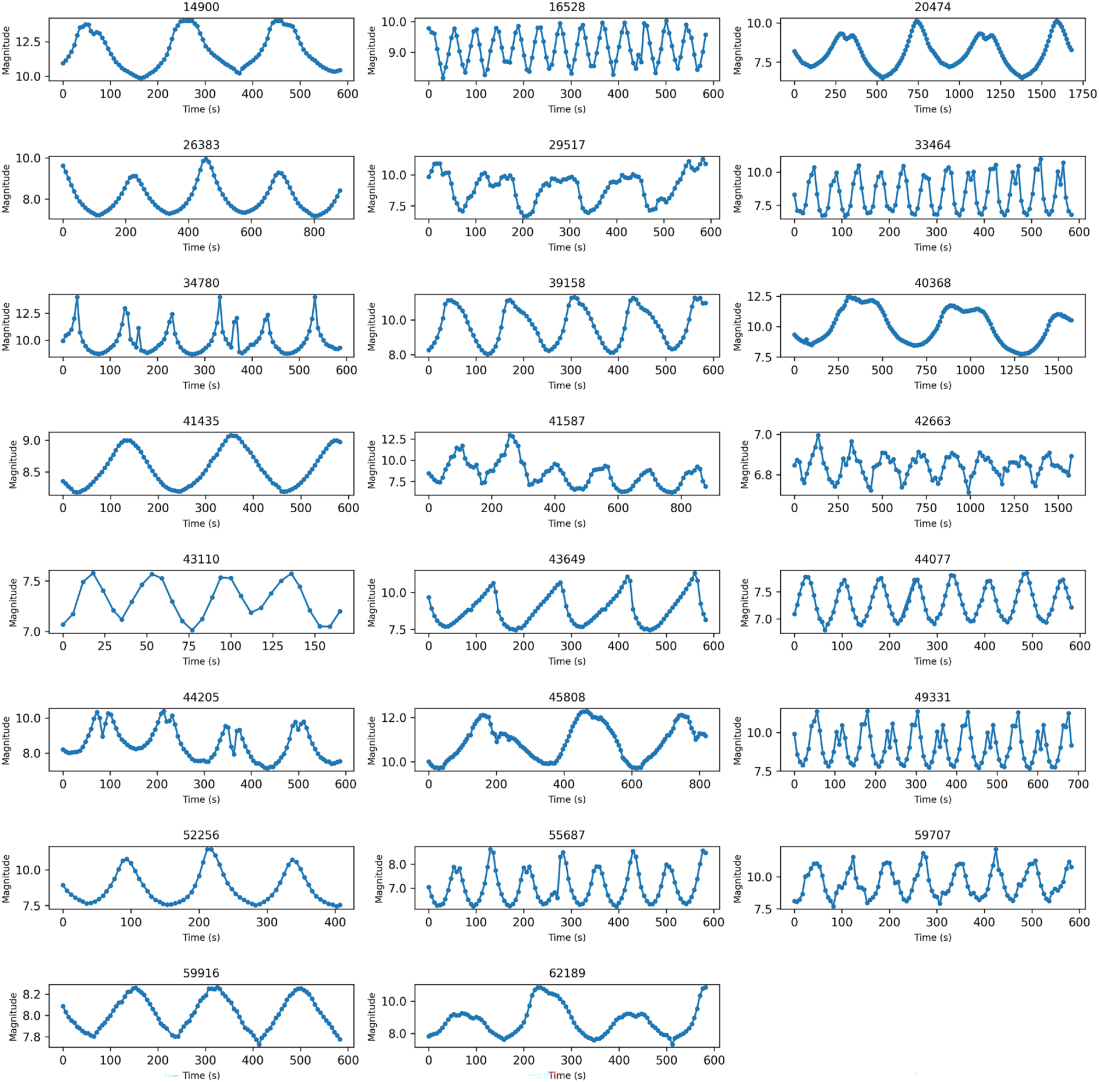
Light curves of all 23 targets, showing that all observations cover sufficient periods with good photometric precision.

After completing the image reduction, we used SExtractor^16^ to extract the positions and fluxes of all sources, including both the targets and the standard stars. Because the observations were conducted in target-tracking mode, the rocket bodies appear as point sources in the images, while background stars are elongated into trails (Fig. 7). By analyzing parameters such as SNR and ellipticity, the rocket bodies can be effectively distinguished from the star trails. Astrometric calibration and source cross-matching were performed on the reference star images. Several high-SNR stars were selected to calculate the photometric zero point and subsequently determine the apparent magnitudes of the targets. Given that the quantum efficiency curve of the QHY600 camera^15^ closely matches that of the Gaia $G_{bp}$ bandpass^17^, we adopted the $G_{bp}$ magnitudes as the reference standard. Figure 4 presents the light curves of all targets listed in Table 1. For subsequent photometric normalization, we used the peak brightness value within each light curve.

### Brightness calibration

During the observations, the targets were at different sun-satellite-observer phase angles, and their slant distances and attitudes varied. To accurately represent the variation in brightness of the CZ-3 series R/B with respect to their time in orbit, the target brightness obtained under different observation conditions must be standardized. This involves calibrating the brightness to a standard slant ranges of 10,000 km, with both the incident and exit angles set to 45°. According to the Lambertian plane model, the apparent magnitude of the reflected light observed from the rocket body can be expressed using the following formula (1)^18^:

1 $$\begin{aligned} \left\{ \begin{aligned}&F(\theta _i, \theta _r) = \frac{I(\theta _i, \theta _r)}{r_o^2} = F_0 \frac{\rho A}{\pi r_o^2} \cos (\theta _i) \cos (\theta _r) \\&m = -26.74 - 2.5 \log _{10} \left[ \frac{\rho A}{\pi r_o^2} \cos (\theta _i) \cos (\theta _r) \right] \end{aligned} \right. \end{aligned}$$

Where *F* is reflected luminous flux, *I* is incident light intensity, $\rho$ is panel reflectance, $F_0$ is incident luminous flux, $\theta _i$is incident angle, $\theta _r$ is emergence angle, *A* is surface area, $r_o$ is the distance between the target and the observation station, *m* is the apparent magnitude of the target.

**Fig. 5:**
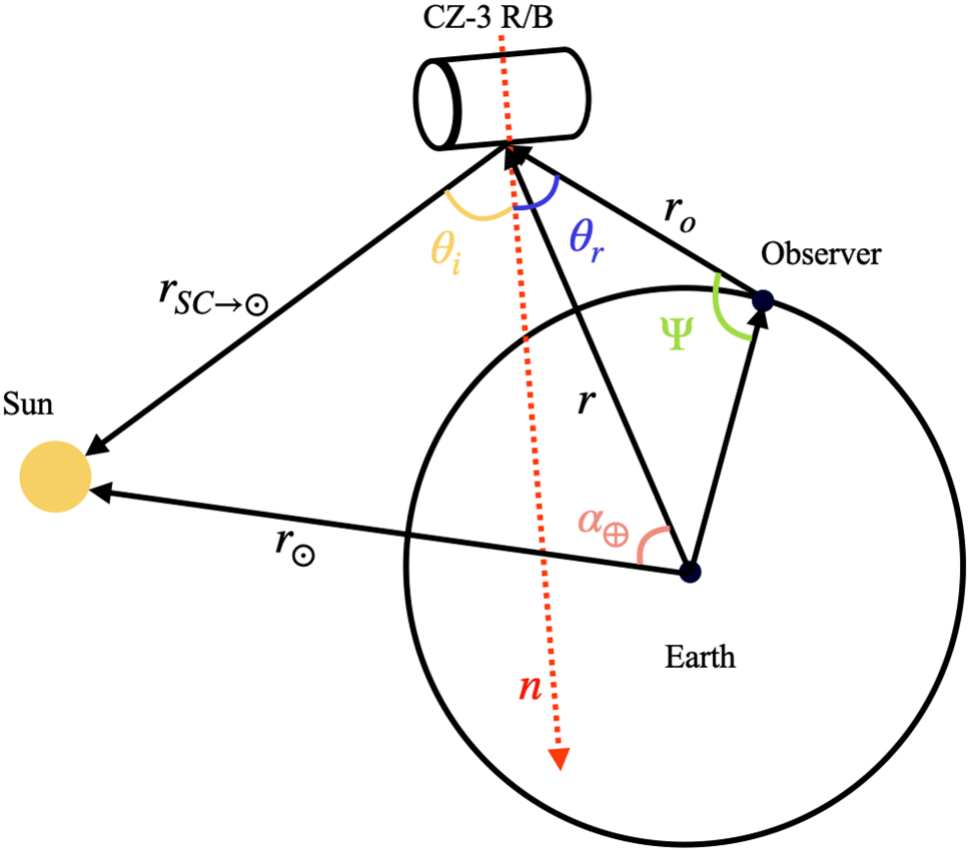
Schematic diagram of the sun-target-observation station geometry at the maximum brightness of the rocket body, where $\theta _i$ = $\theta _r$.

Since the slant range to the targets during observations is generally greater than 10,000 km, the effect of ground-reflected light on the brightness is neglected in this study. The CZ-3 series R/Bs have the same shapes and sizes. After standardizing the calibration for distance, incident angle, and emergence angle, the brightness variations mainly reflect changes in surface reflectivity. Calculating the incidence angle and emergence angle of the target requires obtaining its attitude information. In light-curve-based rotation analysis of space debris, it is generally assumed that in each tumbling cycle there exists a moment when the incident and reflection angles are equal, leading to specular reflection and the peak observed brightness^19–21^. Therefore, in the light curves of the CZ-3 series R/B, the maximum brightness typically corresponds to the moment when the reflective surface faces the largest side of the cylindrical rocket body. At this point, light reflection is predominantly specular, with the incidence angle equal to the emergence angle, as shown in Fig. 5:

In this case, as described in Eq. (2), the calculation of the incidence and exit angles can be simplified by calculating the target-sun vector and the target-observer vector, thereby streamlining the computational process.

2 $$\begin{aligned} \theta _i = \theta _r, \quad \cos (2\theta _i) = \frac{{\bf r}_{SC \rightarrow \odot } \cdot {\bf r}_o}{|{\bf r}_{SC \rightarrow \odot }| |{\bf r}_o|} = 2\cos ^2(\theta _i) - 1 \end{aligned}$$

Since different targets were observed at varying phase angles, their brightness measurements cannot be directly compared. To eliminate the influence of observational geometry, we adopted the assumption defined in Equation (2)—that the condition of equal incidence and emergence angles is satisfied at the moment of maximum brightness—to normalize all brightness values to a reference geometry with both angles set to 45°. Based on the first expression of Eq. (1), and assuming identical target distance, surface area, and reflectance, only the angular dependence of brightness is considered. By combining this with the fundamental assumption that the incidence angle equals the emergence angle during specular reflection, the corresponding angular normalization factor can be derived as follows:

3 $$\begin{aligned} f_{\text {PA}} =F(\frac{\pi }{4},\frac{\pi }{4})/F(\theta _i, \theta _r)= F(\frac{\pi }{4},\frac{\pi }{4})/F(\theta _i, \theta _i) = \left[ 2\cos ^2\left( \theta _i \right) \right] ^{-1} \end{aligned}$$

To correct the brightness of all targets to a range of 10,000 kilometers, the range correction factor $f_{\text {SD}}$ is defined as:

4 $$\begin{aligned} f_{\text {SD}} = \left( \frac{\Vert {\bf r}_o \Vert }{10000} \right) ^2 \end{aligned}$$

Subsequently, using Eq. (5), the brightness of all targets was normalized to a comparable reference condition, corresponding to an incidence angle and emergence angle of 45° and a slant distance of 10,000 km. Under these standardized conditions, since the rocket bodies of the same series share identical structural configurations,the brightness variations among different targets thus reflect changes in their overall albedo, thereby indicating the temporal evolution of their brightness.

5 $$\begin{aligned} F_{\text {cal}} = 10^{\frac{-2x}{5}} \cdot f_{_{\text {PA}}} \cdot f_{_{\text {SD}}}, \quad x \in \{m\} \end{aligned}$$

Finally, the brightness of the oldest target within each rocket series was used as the unit brightness. Using Equation (6), the brightness of all targets was converted into relative brightness, providing a more direct representation of the magnitude of brightness variation.

6 $$\begin{aligned} F_{\text {std}} = \frac{F_{\text {cal}}}{F_{14900}} \end{aligned}$$

Where $F_{\text {cal}}$ is the calibrated brightness, $F_{\text {std}}$ is the standardized brightness of all targets, $F_{14900}$ is the calibrated brightness of the oldest target (NORAD ID :14900), and $\{m\}$ is the set of target apparent magnitudes.

## Results

### Standardized brightness variation curve of CZ-3 series rocket bodies

**Fig. 6:**
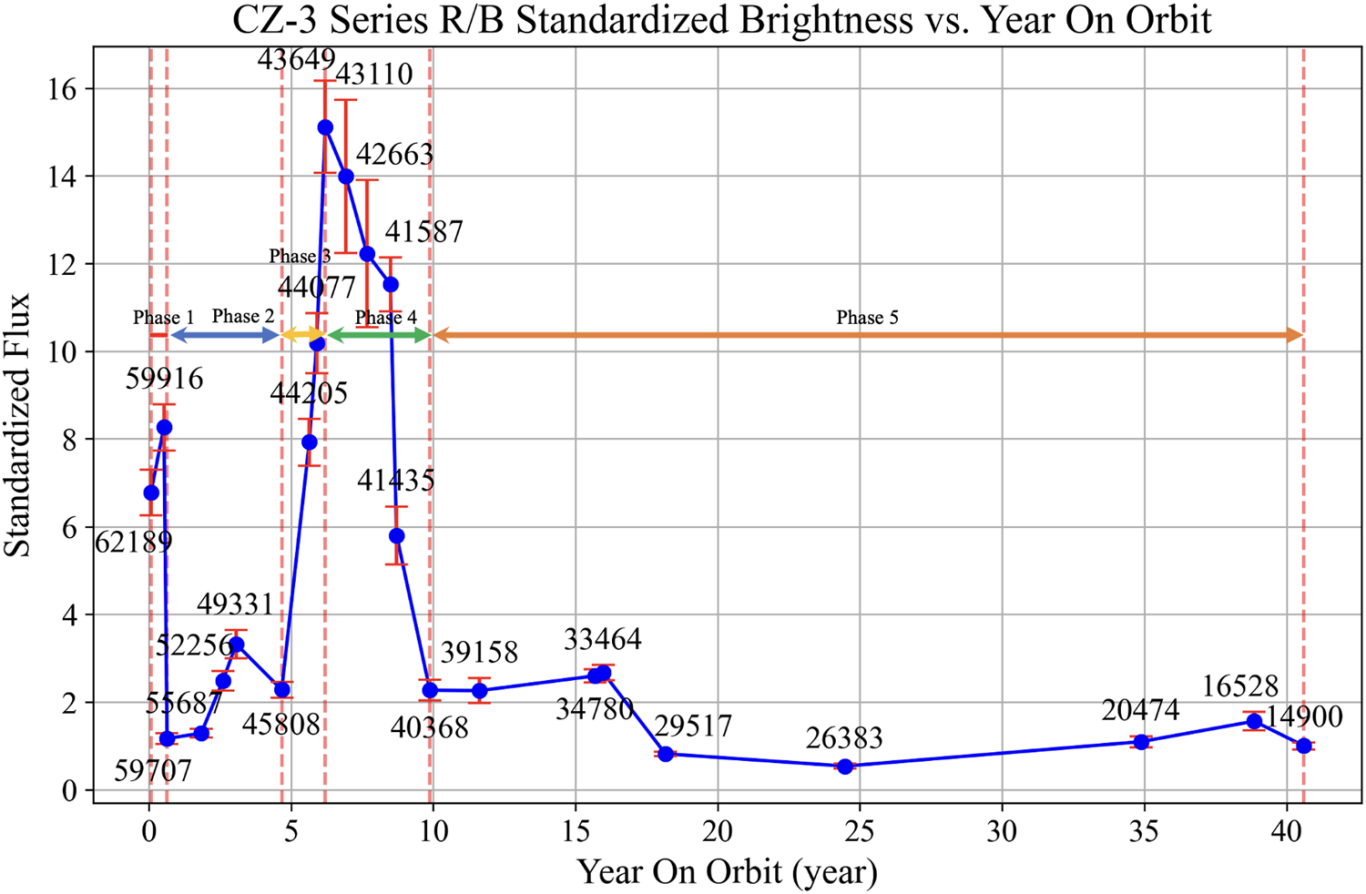
The standardized brightness versus in-orbit duration of the CZ-3 series rocket bodies, which can be roughly divided into 5 stages. Stage 1: During the first year after launch, the brightness decreases at a relatively rapid rate. Stage 2: Between the first and fourth year, the brightness remains stable and have a likely slower increase. Stage 3: Between the 4th and 6th year, the brightness increases drastically and reaches a peak. Stage 4: Between the 6th and 10th year, the brightness rapidly decreases to the level before Stage 3. Stage 5: After the 10th year, the brightness remains low and a slow decrease can likely be seen.

Between November 2024 and March 2025, we observed all of the targets listed in Table 1. After applying the requisite astronomical image reductions to the observation images, we performed differential photometry and flux calibration for the targets. This resulted in the relationship curve between the standardized brightness of the rocket bodies and their ages in orbit (Fig. 6). It is generally accepted that space weathering causes the surface of spacecraft to age and become rougher, leading to a decrease in reflectivity. However, the rocket bodies exhibited an unexpected increase in brightness approximately 5 years after entering orbits. The curve of the CZ-3 series rocket bodies’ brightness versus in-orbit duration does not exhibit a simple monotonic dimming or brightening trend, but instead a non-monotonic pattern with roughly five stages.

In Fig. 6, the uncertainties originate from the magnitudes errors($\sigma _{m_\textrm{cal}}$). These magnitude errors are propagated through Eqs. (5) and (6), and the final uncertainties are obtained using the error propagation formulas as follows:

7 $$\begin{aligned} \sigma _{F_{\text {cal}}} = F_{\text {cal}} \sqrt{ \left( \frac{\ln 10}{2.5}\sigma _{m_\textrm{cal}} \right) ^2 + \left( \frac{\sigma _{f_{\text {PA}}}}{f_{\text {PA}}}\right) ^2 + \left( \frac{\sigma _{f_{\text {SD}}}}{f_{\text {SD}}}\right) ^2 } \end{aligned}$$

8 $$\begin{aligned} \sigma _{F_{\text {std}}} = F_{\text {std}} \sqrt{ \left( \frac{\sigma _{F_{\text {cal}}}}{F_{\text {cal}}}\right) ^2 + \left( \frac{\sigma _{F_{14900}}}{F_{14900}}\right) ^2 } \end{aligned}$$

**Fig. 7:**
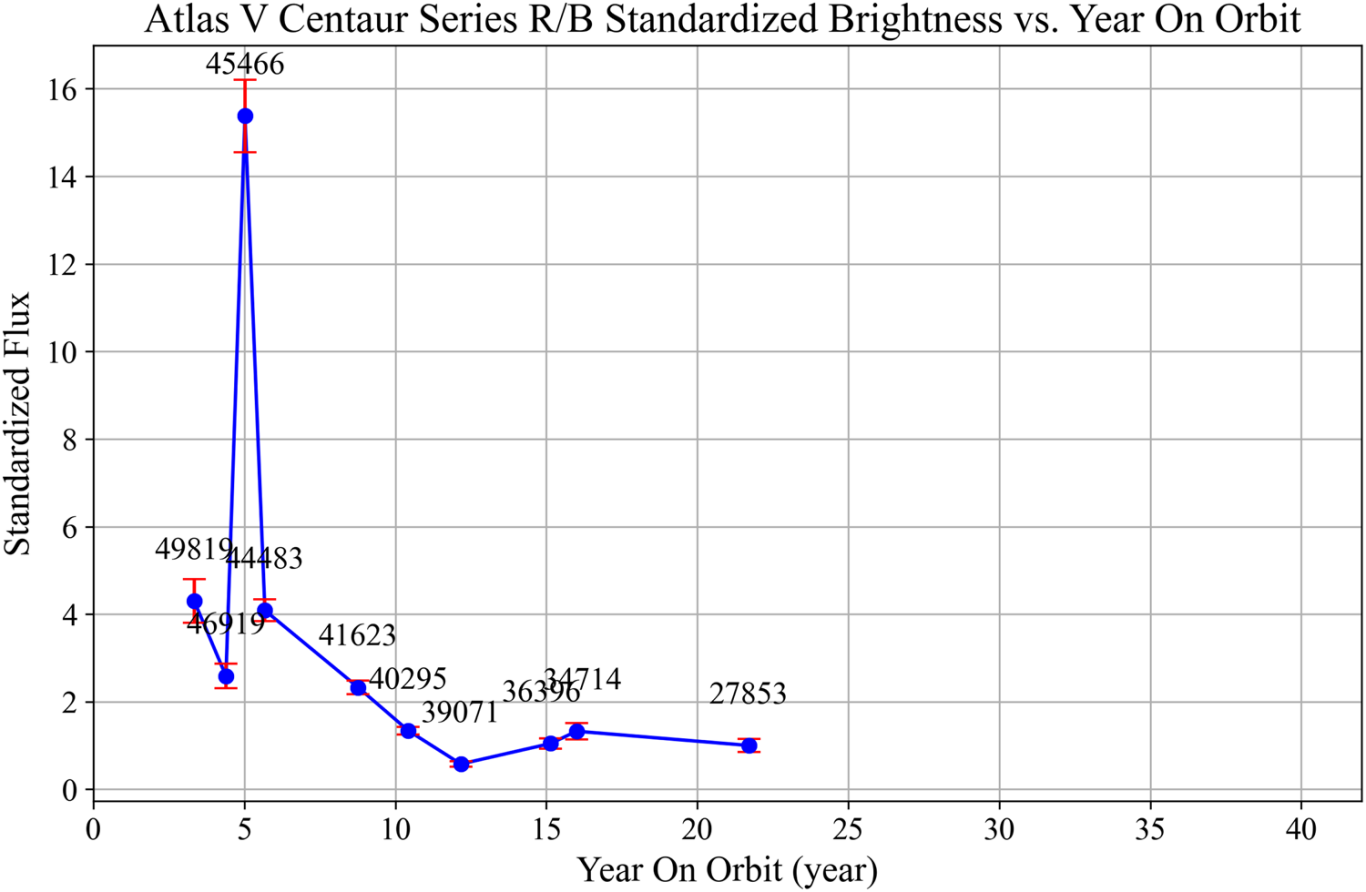
The standardized brightness versus in-orbit duration of the Atlas V Centaur series rocket bodies. The results show that compared to the CZ-3 series, the brightness evolution stages of the Atlas V series have shorter time spans (e.g., the surface material spalling stage occurs about 1 year earlier than for the CZ-3 series rocket bodies), but the overall trend is highly consistent.

Here, the first equation represents the relative uncertainty of the calibrated brightness, the second equation gives the uncertainty of the standardized brightness. Together, these formulas ensure that the error bars in Fig. 6 properly reflect the propagation of uncertainties from the magnitude measurements to the final standardized brightness. The calculation of the uncertainties in Fig. 7 follows the same procedure.

### Standardized brightness variation curve of Atlas V Centaur series rocket bodies

We also obtained the standardized brightness versus in-orbit duration curve for Atlas V Centaur series rocket bodies (Fig. 7). The results indicate that this series rocket bodies’ brightness changes follow very similar patterns to those of the CZ-3 series. However, the changes occur over a shorter timescale. For instance, its brightness-increase stage occurs about 1 year earlier than in the CZ-3 series.

## Discussion

**Fig. 8:**
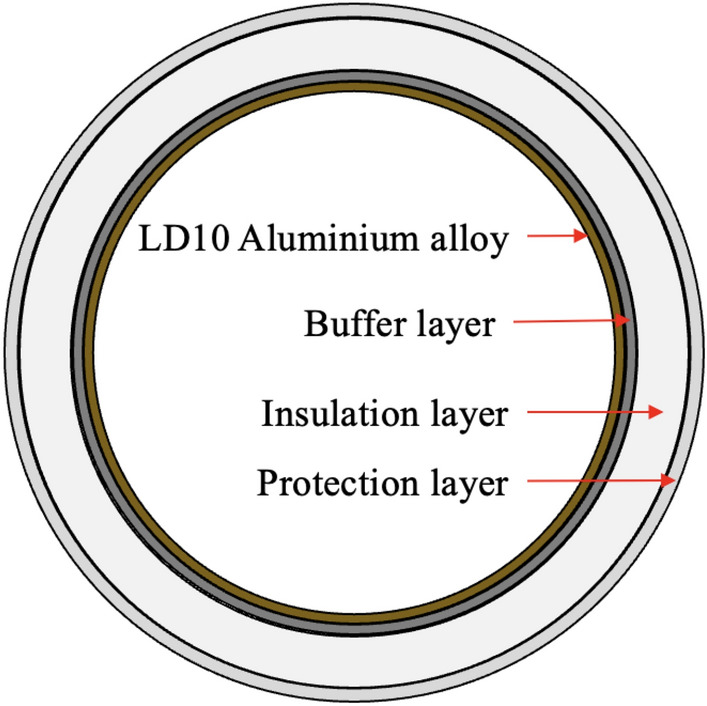
The schematic diagram of the main body and the multi-layered structure encasing the CZ-3 series rocket bodies. The protective layer is typically a high-reflectivity white thermal control coating, with the primary structural material being high-strength fiberglass fabric with high-temperature resistance. The insulation layer is primarily made of polyurethane foam plastic, which serves as thermal insulation. The buffer layer, located between the foam insulation and the metal tank’s outer surface, primarily functions to buffer the delamination tendency caused by the differential linear expansion coefficients between the aluminum alloy tank and the foam plastic, thus maintaining the excellent bonding properties of the sprayed polyurethane foam plastic^23^.

The main body material of the CZ-3 series rocket bodies is LD10 aluminum alloy^22^. The insulation layer on the outer surface of the storage tank uses a multi-layered, sealed wound structure primarily composed of polyurethane foam. It consists of three layers: a buffer layer, an insulation layer, and a protective layer, as shown in Fig. 8.

The third stage of the Long March 3 rocket does not jettison its surface shell during the launch process, consequently, the in-orbit rocket body remains largely intact. Furthermore, because the velocity crossing the Kármán line (100 km) is relatively low (~3 km/s), it generally does not cause the intense thermal damage that is seen during spacecraft reentry into the atmosphere. However, in the harsh space environment, the diverse materials used in the rocket bodies are subjected to a range of challenges. For example, Sharma^24^ reported that the white paint underwent significant degradation when exposed to simulated space conditions, such as ultraviolet radiation, particle radiation, and air-to-vacuum transitions. LEO contains high concentrations of corrosive atomic oxygen, which leads to substantial surface erosion and oxidation reactions on spacecraft materials^25^. These reactions can result in surface corrosion, pitting, material degradation, and even material spalling.

The impact of space weathering on the surface materials of rocket bodies causes gradual aging and degradation, and may also lead to erosion, detachment, etc. Kourtides et al.^26^ confirmed via experiments that the high-temperature environment of space induces embrittlement effects in organic coatings such as Protective Ceramic Coating(PCC), and that the degree of material erosion is positively correlated with the exposure duration. This finding is consistent with Zhou et al.^27^, who reported that fiberglass fabric, the primary material for the protective layer of rocket bodies, also experiences embrittlement and fracture under long-term high temperatures. Furthermore, in a comprehensive study utilizing multiple techniques (radar, optical telescopes, and imaging), McKnight et al.^28^ presented Non-Earth Imaging (NEI) data for a CZ-2D rocket body (NORAD ID: 53878). These NEI images, captured by space-based cameras, accurately depict the target’s in-orbit condition. The observation that the surface material of this CZ-2D R/B in a sun-synchronous orbit has almost completely detached visually demonstrates the cumulative destructive effects of space weathering on spacecraft materials.

Therefore, we propose that the change in the standardized brightness of the rocket bodies with their age in orbit is caused by the following mechanism: During the initial 0–1 years, the white surface coatings degrade due to space weathering effects, including atomic oxygen erosion, high-energy particle irradiation, and exposure to the vacuum environment. This degradation significantly reduces surface reflectivity, causing a noticeable decline in observed brightness. From approximately the first to the fourth year, surface materials were progressively eroded by space weathering, leading to partial shedding of the outer coatings. As a result, the underlying aluminum alloy structure, the primary material of the rocket body, began to be exposed, causing the surface brightness to remain relatively stable with a slight upward trend. After the 4th year, continued space weathering causes the adhesion properties of the rocket body’s surface materials to reach a critical threshold, triggering large-scale shedding. Consequently, between the 4th and 6th years, the brightness rapidly increases as the surface material sheds. The brightness reaches its maximum when the material covering the LD10 aluminum alloy surface is almost completely shed. Subsequently, from the 6th to the 10th year, the LD10 aluminum alloy continues to weather under the influence of ultraviolet radiation, high-energy particle irradiation, atomic oxygen erosion, micrometeoroid impacts, and space debris collisions, which results in a rapid decrease in reflectivity and, therefore, a sharp decline in brightness. Finally, from the 10th to the 40th year, the optical properties of the rocket body’s main material, LD10 aluminum alloy, tend to stabilize under continuous space weathering. In this stage, the surface brightness stabilizes, and although there is a downward trend, the rate of decrease is very slow.

Another potential mechanism may contribute to the pronounced variations in the standardized brightness of the CZ-3 series rocket bodies. After approximately four years in orbit, extensive surface material shedding occurs. The detached fragments may remain partially connected to the main body or be in close proximity, thereby increasing the overall illuminated surface area. This enlargement of the surface area could be one of the key factors driving the sharp rise in standardized brightness observed after the fourth year. Subsequently, after the sixth year, the shed materials gradually separate from the main body and disperse into different spatial positions, reducing the effective illuminated area and consequently leading to a rapid decrease in standardized brightness.

The brightness evolution pattern of the Atlas V rocket bodies closely resembles that of the CZ-3 series. However, each stage occurs over a shorter timescale. For example, the surface material shedding stage occurs approximately one year earlier than in the CZ-3 series. Given the largely similar structure of these two rocket families^29^, the earlier timing of surface material shedding in the Atlas V series may be attributed to the relatively lower weathering resistance of its surface protective materials. These observations suggest that space-weathering-induced corrosion and delamination of surface materials may be a common phenomena among multi-layer structured rocket bodies in GTO.

We also explored the destination of these shed surface materials. Based on the physical properties of the polyurethane foam plastic on the rocket body surface, we speculate that it may shed in flake-like forms under space weathering. This spalling process can maintain relatively good geometric integrity of the shed material, producing debris with a HAMR. Such debris can evolve along two orbital paths. One path, as pointed out by Rosengren^30^, is that HAMR objects in high Earth orbit exhibit periodic variations in orbital inclination and eccentricity under the influence of solar radiation pressure, Earth’s oblateness, and lunisolar perturbations. The maximum amplitude of orbital inclination oscillation is between 25° and 35°, with a long-term oscillation period of about 22 years. Schildknecht et al. detected a family of uncorrelated space debris in GEO and GTO orbits^11,12^. These objects have a very high AMR (1–28 m^2^/kg) and their orbital inclinations are concentrated between 0°–15°, significantly different from the characteristics of usual fragmentation debris. According to Rosengren’s results^30^, the surface material shed from Atlas V series rocket bodies (with inclinations mainly between 0°–30°) and CZ-3 series rocket bodies (inclinations mainly between 20-30°) could be one of the sources of these observed uncorrelated objects. The other evolutionary path is re-entry and ablation due to atmospheric drag. Studies by Pazderová et al.^31^ and Pardini et al.^32^ indicate that CZ-3 series rocket bodies in low-altitude regions (perigee< 300 km) are significantly affected by atmospheric drag, leading to orbital decay and eventual re-entry. After separating from the parent body, shed debris may rapidly re-enter the atmosphere due to atmospheric drag, making it difficult to remain in GTO for extended periods. Most of the space debris formed by the spalling of rocket body surface material should have their orbits affected by the combined action of these two factors.

## Conclusion

In this paper, we used the 80-cm aperture MEET of PMO to conduct optical observations of 23 CZ-3 series rocket bodies located in GTO. We discovered a multi-stage evolutionary pattern in their standardized brightness with respect to their in-orbit duration. Based on our understanding of the rocket surface materials, we propose that the changes in the rocket body brightness correspond to five stages of evolution in its surface material. This multi-stage long-term photometric change was also validated by the observation of another series of rocket bodies, the Atlas V series, indicating that this pattern is likely a common phenomenon of the long-term evolution of rocket body surface materials.  The shed polyurethane foam material possesses a HAMR and is likely a source of uncatalogued HAMR debris in GTO. Based on this model, we will conduct multi-band photometric observations, integrating the target’s color indices and brightness information with the aim of predicting the on-orbit duration of unknown objects, thereby gaining multi-dimensional information for debris identification, cataloguing, and source tracing.

## Data Availability

The datasets generated during and/or analyzed during the current study are available from the corresponding author on reasonable request.

## Code availability

The code for the image reduction, photometry and brightness calibration of the datasets written in Python is available on reasonable request.
